# Deciphering perivascular macrophages and microglia in the retinal ganglion cell layers

**DOI:** 10.3389/fcell.2024.1368021

**Published:** 2024-03-26

**Authors:** Jehwi Jeon, Yong Soo Park, Sang-Hoon Kim, Eunji Kong, Jay Kim, Jee Myung Yang, Joo Yong Lee, You-Me Kim, In-Beom Kim, Pilhan Kim

**Affiliations:** ^1^ Graduate School of Medical Science and Engineering, Korea Advanced Institute of Science and Technology (KAIST), Daejeon, Republic of Korea; ^2^ KI for Health Science and Technology (KIHST), Korea Advanced Institute of Science and Technology (KAIST), Daejeon, Republic of Korea; ^3^ Department of Anatomy, College of Medicine, The Catholic University of Korea, Seoul, Republic of Korea; ^4^ Institute for Basic Science, Daejeon, Republic of Korea; ^5^ Department of Neuroscience, Columbia University, New York, NY, United States; ^6^ Asan Medical Center, University of Ulsan College of Medicine, Seoul, Republic of Korea

**Keywords:** retinal microglia, superficial microglia, perivascular macrophage, central nervous system border-associated macrophage, CD86^+^ macrophage

## Abstract

**Introduction:** The classically defined two retinal microglia layers are distributed in inner and outer plexiform layers. Although there are some reports that retinal microglia are also superficially located around the ganglion cell layer (GCL) in contact with the vitreous, there has been a lack of detailed descriptions and not fully understood yet.

**Methods:** We visualized the microglial layers by using CX3CR1-GFP (C57BL6) transgenic mice with both healthy and disease conditions including NaIO3-induced retinal degeneration models and IRBP-induced auto-immune uveitis models.

**Result:** We found the GCL microglia has two subsets; peripheral (pph) microglia located on the retinal parenchyma and BAM (CNS Border Associated Macrophage) which have a special stretched phenotype only located on the surface of large retinal veins. First, in the pph microglia subset, but not in BAM, Galectin-3 and LYVE1 are focally expressed. However, LYVE1 is specifically expressed in the amoeboid or transition forms, except the typical dendritic morphology in the pph microglia. Second, BAM is tightly attached to the surface of the retinal veins and has similar morphology patterns in both the healthy and disease conditions. CD86^+^ BAM has a longer process which vertically passes the proximal retinal veins. Our data helps decipher the basic anatomy and pathophysiology of the retinal microglia in the GCL.

**Discussion:** Our data helps decipher the basic anatomy and pathophysiology of the retinal microglia in the GCL.

## 1 Introduction

Intravitreal injection methods are one of the major procedures in ophthalmology, and newly developed drugs are continuously being invented for them (Tadayoni et al., 2021; Heier et al., 2022; Wykoff et al., 2022). To properly understand these newly developed drugs, it is important to understand various characteristics of the vitreous, including hyaluronic acid coiling, remodeling, liquefaction, and metabolism of the interface between the vitreous and retina. The inside of the eye is filled with the vitreous, which mainly consists of type II collagen and hyaluronic acids. It is also known that aggregation and conformational changes in the vitreous may decrease the efficacy of protein drugs and may also have immunological consequences (Del Amo et al., 2017). However, studies on the immunological reactions between the vitreous and retina remain insufficient. On the surface of the retina facing the vitreous, there are some immunological cells, such as the retinal microglia. However, layers of the retinal microglia are classically defined into two anatomically separated layers: the inner plexiform layer (IPL) and outer plexiform layer (OPL) under a healthy condition (O'Koren et al., 2019; Beguier et al., 2020). The IPL contains neuronal synapses between the retinal ganglion cell and bipolar cell, and the OPL contains synapses between the bipolar cell and photoreceptor cells, such as cone and rod cells. It is well known that the microglia play a key role in synapse pruning (Paolicelli et al., 2011; Lewis, 2021). It seems natural, then, that the retinal microglia also live in the plexiform layers containing many synapses. Although some reports indicate the superficial microglial layer (Madeira et al., 2015; Choi et al., 2021), the characteristics and roles of the microglia on the ganglion cell layer are yet to be clearly verified.

Central nervous system (CNS) border-associated macrophages (BAMs) include cells residing in the perivascular spaces of brain vessels, lining other tissues such as meninges, and the choroid plexus (Dermitzakis et al., 2023). Recently, perivascular macrophages in the brain have received significant attention (Masuda et al., 2022; Siret et al., 2022). It seems that BAMs carry out scavenger functions and are fully competent to present antigens to lymphocytes (Mrdjen et al., 2018; Kim et al., 2021). However, in the healthy retina, antigen-presenting cells (APCs) are still unclear, even though the microglia conditionally express MHC II molecules in blood–retinal barrier (BRB) disrupted states. Furthermore, the APC of the interface between the vitreous and retina is yet to be revealed.

Herein, we successfully visualize the details of the superficial microglial layer located in the GCL with specific markers such as LYVE1, galectin-3, and CD86 under healthy conditions. In particular, LYVE1 and galectin-3 are only located in the peripheral retina, and CD86 is expressed in some parts of the BAM at the proximal retinal veins lining the venous surface.

## 2 Materials and methods

### 2.1 Animal models

All animal experiments were approved by the Institutional Animal Care and Use Committee of the Korea Advanced Institute of Science and Technology (KAIST) (approval No. KA 2021-056). All animals were treated, maintained, and euthanized according to the policies specified in the ARVO Statement for the Use of Animals in Ophthalmic and Vision Research. Mice were housed and bred in an institutional animal facility in KAIST. All mice were individually housed in ventilated and temperature- and humidity-controlled cages (22.5°C and 52.5%) under a 12/12-h light/dark cycle and provided with a standard diet and water *ad libitum*. For experimental use, C57B6/N mice were purchased from Orient Bio (Suwon, Korea). CX3CR1-GFP mice were purchased from the Jackson Laboratory (#005582, Bar Harbor, Maine, United States). CX3CR1-CreERT2 (#020940), LSL-Ai14 (# 007914), and CSF1R-GFP (#018549) mice were also purchased from the Jackson Laboratory and cross-bred to create a conditional CX3CR1-RFP reporter mouse. TLR4 K/O mice were generously provided by Professor Y-M Kim (KAIST, Daejeon, Korea). LoxP CD86 loxP tdTomato mice were created by Cyagen (Santa Clara, California, United States). This conditional knock-in model was created by CRISPR/Cas-mediated genome engineering. The guide RNA sequences used for vector manufacturing are as follows:

gRNA-A1 (ACC​ATC​ATC​ATA​ATT​GCT​ACA​GG)

gRNA-A2 (GGT​TAG​GCT​AGC​TCT​CTA​TGG​GG)

gRNA-B1 (GCC​TGT​AGC​AAT​TAT​GAT​GAT​GG)

gRNA-B2 (AGG​TTA​GGC​TAG​CTC​TCT​AT-GGG)

The inserted gene location is in the chromosome 16:36,424,231-36,486,443 reverse strand, and the sequence is

5′ATA​ACT​TCG​TAT​AGC​ATA​CAT​TAT​ACG​AAG​TTA​TTG​GTA​GTA​CTC​CCA​GTC​ATA​GCT​GTC​CCT​CTT​CTC​TTA​TGG​AGA​TC3ʹ.

To induce RPE cell degeneration and induce microglial activation, sodium iodate (NaIO_3_, 50 mg/kg, stock no: S4007, Sigma-Aldrich, St. Louis, Montreal, United States) was injected via the peritoneum in a 0.05% acetic acid solution (stock no: A6283, Sigma-Aldrich). Analysis was done according to each experiment. To induce inter-photoreceptor retinoid-binding protein (IRBP)-induced autoimmune uveitis, IRBP 1–20 (GenScript, Piscataway, New Jersey, United States) was emulsified in complete Freund’s adjuvant (CFA, 1:1 vol/vol; Sigma-Aldrich) and systemically administered intramuscularly (0.2  mL emulsification per mouse, at the tail base) as previously described (Yang et al., 2022).

### 2.2 Sample preparation for histological analyses

The mice were euthanized using a CO_2_ chamber. Both whole eyeballs with optic nerves were carefully harvested by using forceps without tearing and immersed in 1% paraformaldehyde solution overnight for the fixation of the entire tissue. After washing the fixed eyeballs with PBS, they were placed in a small cell culture dish under a stereoscopic microscope. A linear incision was made using a No. 11 blade at the center of the cornea. Subsequently, a circular incision was made through the limbus using iris scissors, and the cornea was detached. The crystalline lens, ciliary body, and iris were stripped off, and the optic nerve was gently cut while avoiding tangential traction damage in the retina. The vitreous was carefully and completely removed from the retina. The neuro-retinal/ONH fraction and the choroid/RPE fraction were divided through subretinal space dissection. Retinal tissues were trimmed to make a four-leaf clover shape for visualizing *en face* flat-mounted images. Vertical sectioning of the whole layers in the eye and optic nerve was acquired from the overnight fixed samples as described above. However, these samples were placed on 3.5% agarose gel (SeaKem^®^ LE Agarose, #50004, Lonza, Basel, Switzerland) for embedding. The embedded samples were sliced using a vibratome (20∼100-µm thickness, 0.28 mm/s, 1.5-mm width, VT1200S, Leica, Wetzlar, Germany). These processed samples (flat-mounted and sliced) were blocked with 5% normal goat serum (NS02L-1ML, Sigma-Aldrich) or bovine serum albumin (BSA, A7030-10G, Sigma-Aldrich) in PBST (0.3% Triton X-100 in PBS) and incubated overnight at 4°C with the following primary antibodies (1:200): anti-CD31 (PECAM, rat, #553708, BD Biosciences, Franklin Lakes, New Jersey, United States, hamster, 2H8 clone, MA3105, Thermo Fisher), anti-Iba1 (rabbit, #PA5-27436, Thermo Fisher Scientific, Waltham, Massachusetts, United States), anti-galectin-3 (rabbit, #ab209344, Abcam Inc., Cambridge, United Kingdom), anti-LYVE-1 (rabbit, #ab218535, Abcam Inc.), anti-CD86 (rabbit, #ab242142, Abcam Inc.), and anti-CD44 (rabbit, #ab243894, Abcam Inc.). After washing 8 times in 0.3% PBST, the samples were incubated for 2 h at RT in a shaker with species-specific Alexa Fluor-coupled secondary antibodies (1:250) in 0.3%. PBST solution (goat anti-rat A555 secondary antibody: A21434, Thermo Fisher; goat anti-rat A647 secondary antibody: ab150167, Abcam; goat anti-rabbit A555: A32732; goat anti-rabbit A647: A32733, Thermo Fisher; and goat anti-hamster A488: A21110, Thermo Fisher). All the antibodies used in this study were validated for the species and applications by the indicated manufacturers. Subsequently, the samples were washed four times in 0.3% PBST and then four times in PBS and placed on a glass slide with a mounting medium (VECTASHIELD^®^ Anti-fade Mounting Medium, H-1000-10, Vector Laboratories, Burlingame, California, United States).

### 2.3 Imaging and morphometric analyses

Immunofluorescence images were acquired using a commercial confocal microscope (IVM-FS system, IVIM Technology, Daejeon, Korea). ImageJ software (NIH) was used for the acquisition and processing of images. Confocal images of whole-mount samples were maximum intensity projections of tiled z-stack images taken at 1-μm intervals through the entire thickness of tissues, which were all taken at a resolution of 2,048 ×2,048 pixels with the commercial objectives (PlanApoλ20X, 0.75NA; Nikon Corporation, Tokyo, Japan, LUMFLN60XW, 1.1 NA, water; Olympus, Tokyo, Japan) with multichannel scanning in frame. Then, 3D reconstruction and cut images were created from z-stack confocal images using Imaris software (ver 9.02, Bitplane, Belfast, United Kingdom). The interglial distance was calculated by utilizing z-stack imaging of the whole-retinal sample using ImageJ. Center points of the soma on each layer were selected as the standard point for quantifications. The number of CX3CR1 cells and amoeboid-form microglia was semi-automatically quantified with more than 12.5 μm of the estimated X-Y diameter in the “spot” function in Imaris. A filter type was selected as “quality” (lower threshold: 4.0; upper threshold: 15.0 in ROI). CX3CR1-GFP+ cells were regarded as amoeboid-shaped when they showed one of the following features: retraction of their processes and an increased long axis of the soma above 22.5 μm in Imaris. The CX3CR1/CD31 overlapped area ratio was calculated by utilizing the “surface” function of Imaris commercial image analysis software. The “surface detail” value of the area was determined as 1 μm, and the minimum and maximum cut-off values were set to 20.0 and 30.0, respectively. Then, the co-localized area was divided by the total CD31 area. To quantify the morphological type of the LYVE1+ cells, subtypes were divided into three categories: dendritic form, transition form, and amoeboid form. CX3CR1 cells, which have more than four processes with LYVE1 expression, were considered dendritic forms. The amoeboid form was considered when they did not have a process and have the LYVE1+/CX3CR+ expression in the soma. Veins were classified morphologically as having a thick diameter and CD31 staining in a net pattern with discontinuous parts inside, while arteries were thin in diameter and had straight CD31 staining.

### 2.4 Single-cell RNA sequencing analysis

Single-cell RNA sequencing (scRNA-seq) data (10× genomics) were retrospectively collected from the Gene Expression Omnibus (GEO; GSE137537 (Menon et al., 2019) for the whole human eye and GSE121081 (Ronning et al., 2019) for the mouse microglia). ScRNA-seq data processing and analysis were implemented using the Seurat v.4.1.0 R package. Quality control metrics were designed for discarding low-quality cells (<200 genes/cell, >4,500 RNA genes/cell, and >5% mitochondrial genes). Log normalization was done with “scale factor” = 10,000. The top 10 genes were used to find different cell clusters using the graph-based clustering method with the “FindClusters” function (resolution = 0.5). A principal component assay was performed for the integrated data matrix to reduce its dimensionality. Then, major cell clusters were annotated using the known cell-type marker genes and visualized using T-distributed stochastic neighborhood embedding (t-SNE) and a Uniform Manifold Approximation and Projection (UMAP) scatter plot. In the human data, ligand–receptor interaction data were acquired using the “netAnalysis_signalingRole_network” function of the CellChat packages from the CellChatDB (http://www.cellchat.org/cellchatdb). In the mouse data, microglial cells were filtered by the CCR2 population to remove the monocytes.

Human genes used for clustering major cell types are as follows:

16 (cluster number) = microglia (CX3CR1 and P2RY2), 15 = endothelial cells (VWF and CLDN5), 2 = retinal ganglion cells (NEFM and FABP3), 5,10 = bipolar cells (TRPM1 and TRNP1), 14 = horizontal cell (LHX1), 13 = amacrine cells (GAD1 and CARTPT), 8 = mg-associated bipolar cells (SLC38A1, CHN2, PRKCB, and THSD7A), 1,6,7,9,12 = Muller cells (GLUL, WIF1, NES, VIM, and CD44), 3 = cone cells (PDE6H), and 11 = rod cells (PDE6G and RHO)

### 2.5 Cell isolation and staining protocols for flow cytometry

Tissue samples were harvested from the neuro-retina and choroid fraction in cold RPMI 1640 solution in the same manner described above in Section 2.2 for flat-mounted images. However, a fresh sample was finely chopped using iris scissors for 90 s in a glass bottle at 4°C. Neuro-retina fraction samples were digested with a mixture solution of 0.4 mg/mL DNase (Roche, Basel, Basel-Stadt, Switzerland) and 1.5 mg/mL collagenase IV 1 (Worthington Biochemical, Lakewood, New Jersey, United States). The digested samples were incubated in media for 45 min at 37°C and stirred before being passed through the Falcon^®^ 40-µm cell strainer (#352340, Corning Inc., NY, United States). Cell suspensions were stained with Ghost Dye™ Violet 510 (13-0870-T100, Tonbo Biosciences, San Diego, California, United States) for live cell staining and then stained with the following antibodies: CD45.2 (CD45.2 monoclonal antibody (104), #12-0454-82, PE, eBioscience), CD11b (PE-Cy™7 rat anti-CD11b, #552850, BD), CD4/8/B220 (VioletFluor™ 450 anti-mouse CD4 (RM4-5), # 75-0042-U100, Tonbo; Pacific Blue™ rat anti-mouse CD8a, #558106, BD; and rat anti-mouse CD45R/B220, #562922, BD), CD86 (APC/Cyanine7 anti-mouse CD86 antibody, #105029, BioLegend, California, United States), and TNF-α (Brilliant Violet 605™ anti-mouse TNF-α antibody, #506329, BioLegend). A measure of 0.5% saponin (SAE0073, Sigma-Aldrich) was used for the cell permeating solution. Samples were acquired on a FACSCalibur system (BD Biosciences). All data were analyzed using FlowJo (Tree Star, Oregon, United States).

### 2.6 Statistical analysis and reproducibility

Statistical difference was determined by a two-tailed non-parametric Mann–Whitney U test and repeated ANOVA test. Statistical analysis was performed using IBM SPSS Statistics 26.0 (SPSS, Inc., Chicago, IL, United States). Statistical significance was set at *p*-values less than 0.05. Statistical graphs were drawn using the Jupyter Notebook (Python Software Foundation, Wilmington, Delaware, United States).

## 3 Results

### 3.1 The superficial microglial layer is located around the GCL and superficial capillary plexus

**FIGURE 1:**
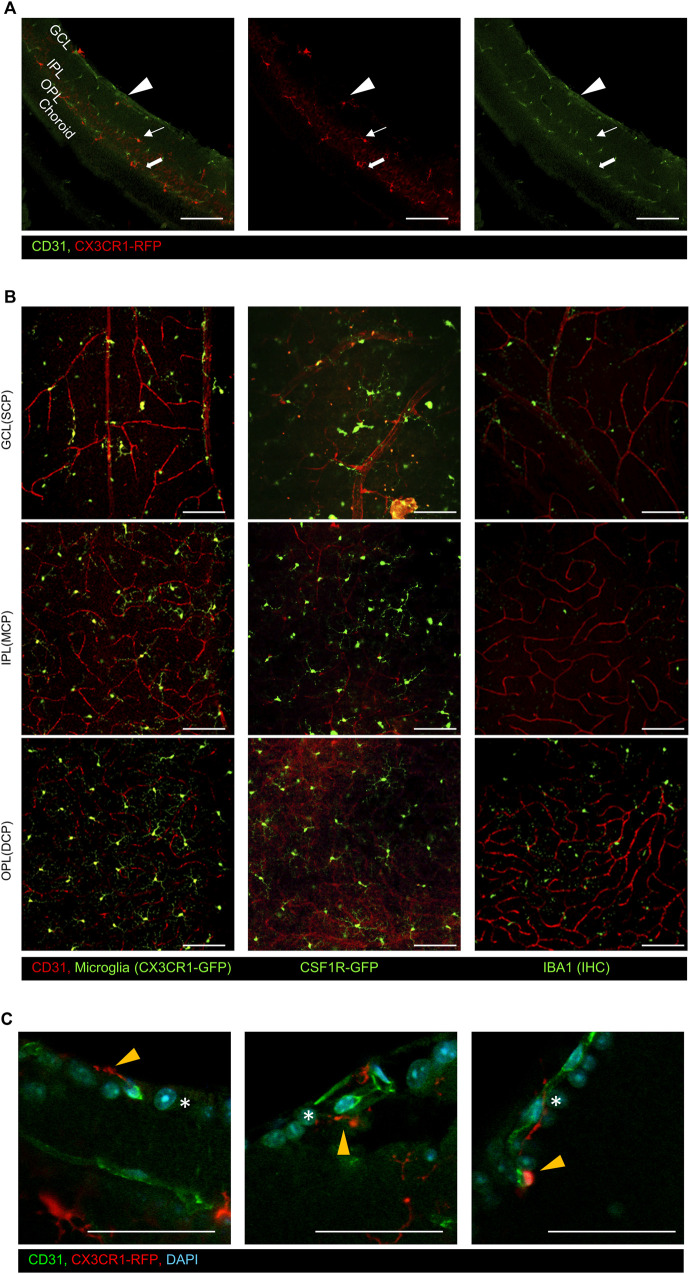
Representative immunostaining images of the CD31 antibody on the retinal surface using CX3CR1 reporter mice. **(A)** Vertical images show that CX3CR1-RFP+ cells were distributed alongside the CD31+ capillary layers. The arrowhead indicates the superficial capillary plexus, the arrow indicates the intermediate capillary plexus, and the thick arrow indicates the deep capillary plexus, **(B)** representative flat-mounted retinal images in the ganglion cell layer (GCL), inner plexiform layer (IPL), and outer plexiform layer (OPL) with common microglial reporter mice and markers (CX3CR1-GFP, CSF1R-GFP, and Iba1). **(C)** Magnified images of the GCL showing that microglia are distributed around the retinal ganglion cells by using CX3CR1-RFP mice. The orange arrowhead indicates GCL microglia, and asterisks indicate the retinal ganglion cells. Scale bar: 100 μm.

There are three different capillary plexuses in the retina: superficial (SCP), intermediated (MCP), and deep capillary plexuses (DCP) (Garrity et al., 2017; Scharf et al., 2021). These vessels are located in the GCL, IPL, and OPL, respectively. The histological analysis of the vertical section of CX3CR1-Ai14, which is microglial RFP reporter mice, shows that the microglia responded to each capillary plexus. CX3CR1-Ai14+ cells reside alongside the capillary plexus stained by CD31, as shown in Figure 1A. We also validated the distribution of the retinal microglia on each layer with an *en face* view by analyzing other commonly used microglial reporter mice such as CX3CR1-GFP, CSF1R-GFP, and IBA1 antibody, as shown in Figure 1B. It is commonly known that microglia are located in the neuronal synapse area around the MCP and DCP (MCP: bipolar to ganglion cells; DCP: photoreceptor to bipolar cells). However, microglia in the SCP are not as much of a concern as other microglia. Even though there are no neuronal synapses in the GCL, microglia in the SCP are vertically distributed within the nerve fiber layer and ganglion cell layer, as shown in Figure 1C. We hypothesized that non-synaptic microglia on the superficial GCL have a different function from other synaptic microglia.

### 3.2 Changes in the inter-microglial gap under the aging and disease condition

**FIGURE 2:**
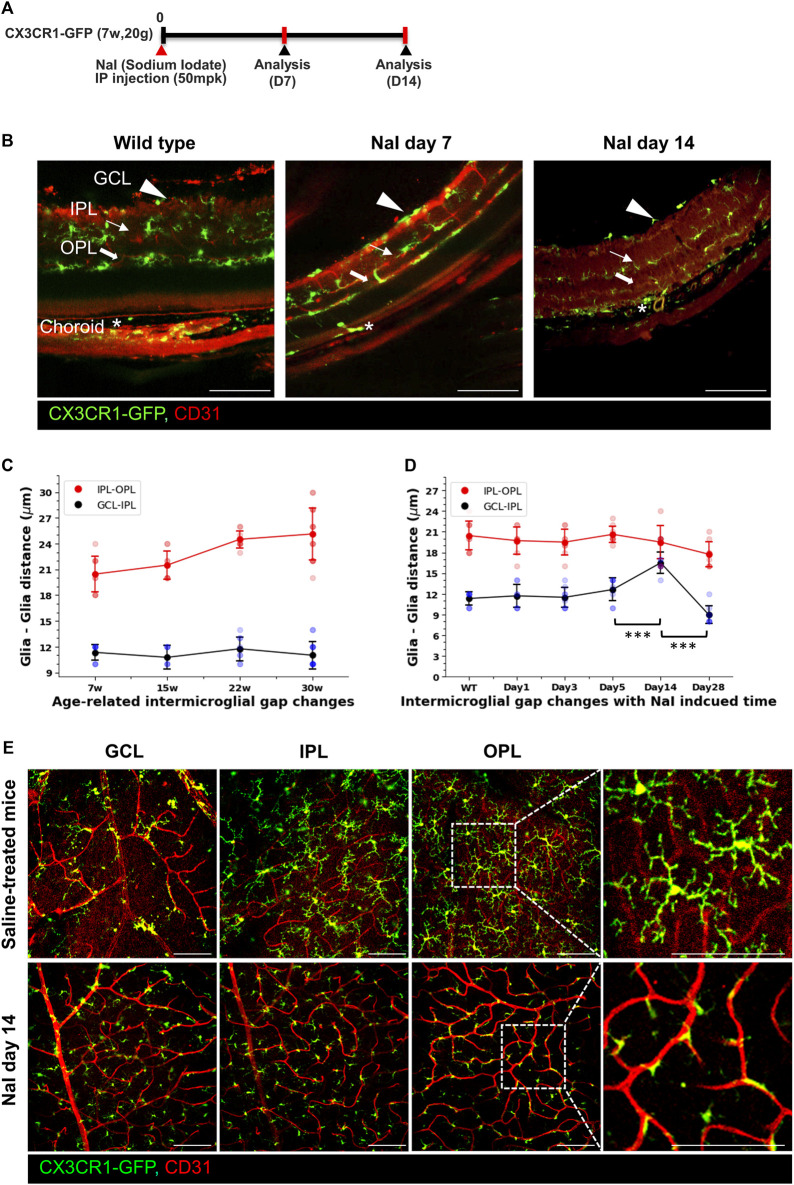
Visualization of the inter-glial distances in the healthy mouse and increased inter-glial distances by the sodium iodate (NaIO_3_)-induced RPE degeneration models. **(A)** Schematic timelines for the NaIO_3_ (50 mpk)-induced RPE degeneration models. **(B)** Vertical images showing that borderlines of the inter-glial distances between the GCL and IPL in the healthy state are obscure and prominently distinguished on days 7 and 14 after NaIO_3_ modeling. **(C)** Linear and scatter graphs of the inter-glial distances (GCL to IPL: blue line; IPL to OPL: red line) from 7 to ∼30 weeks old, showing that inter-glial distances from the GCL to IPL are relatively stable with aging. *n* = 9/8/8/16. **(D)** Linear and scatter graphs of the inter-glial distances during NaIO_3_-induced RPE degeneration progress, showing that the inter-glial gap from the GCL to IPL abruptly increased on day 14 after modeling. *n* = 9/7/8/8/8/8. **(E)** Representative flat-mounted retinal images of the control and NaIO_3_ day-14 mouse, showing that CX3CR1-GFP+ cells adhered more to the vascular surface in the disease model. Scale bar: 100 μm; *p < 0.05, **p < 0.005, and ***p < 0.001.

We believe that many researchers have not focused on GCL microglia for three major reasons. First, microglia have been believed to dominantly function as creating neuronal environments in the CNS (Li and Barres, 2018; Thion et al., 2018). Second, the exact roles of the GCL microglia are yet to be revealed. Lastly, long processes of the IPL microglia make it difficult to distinguish between GCL and IPL microglia. To create a larger inter-glial gap for prominent visualization, the NaIO_3_ (5014 mpk)-induced RPE degeneration models were used (Kannan and Hinton, 2014; Chowers et al., 2017). These models primarily induce RPE degeneration, causing secondary changes in the photoreceptor cells and microglia. These mouse models were investigated for 28 days after NaIO_3_ injection, as shown in Figure 2A. Under a healthy condition, there are many processes of microglia, making it difficult to distinguish the inter-glial gap between the IPL and GCL. However, these microglia are prominently and significantly separated at 14 days after NaIO_3_ injection, and each microglial layer is visualized and divided into three layers as the RPE degeneration progresses, as shown in Figure 2B. Next, to measure the inter-glial distance from the GCL to IPL and IPL to OPL of the aging wild-type mice, CX3CR1-GFP mice between 7 and 3014 weeks old were quantified. Although the axial length of the rodent eyes is elongated during aging, as described in a previous study (Jeon et al., 2022), the GCL-to-IPL distance is similar (7 w: 11.33 ± 0.94, 15 w: 10.75 ± 1.39, 22 w: 11.75 ± 1.39, and 30 w: 11.0 ± 1.58 μm, *n* = 9/8/8/16) between 7 and 30 weeks old, as shown in Figure 2C. However, the GCL-to-IPL distance prominently increases up to 16.5 ± 1.58 μm (*n* = 8) on day 14 after NaIO_3_ injection (Figure 2D and Supplementary Video S1). The microglia of each layer adhere to the vascular walls of the capillary plexus during disease progression with an increased total of CX3CR1+ cells (the total number of CX3CR1 cells at the NFL, IPL, and OPL in the control vs. day-14 groups (*n* = 16/12); NFL: 0.69 ± 0.12 vs. 1.28 ± 0.34, IPL: 0.94 ± 0.10 vs. 1.54 ± 0.41, and OPL: 1.16 ± 0.12 vs. 2.24 ± 0.60 [10^-4^/µm^2^], all groups of the *p*-value < 0.001), as shown in Figure 2E, Supplementary Figures S1, S2. Therefore, the inter-glial distance is prominently visualized, and GCL microglia can become distinguishable. In addition, other disease models such as IRBP-induced autoimmune uveitis have phenotypes similar to the NaIO_3_ model (Supplementary Figure S3), as shown in a previous article (Yang et al., 2022). Under some inflammatory conditions, the microglia in each layer seem to be adhered to near the capillary plexus, and the microglia of each layer can easily be revealed.

### 3.3 Subset of the GCL microglia; peripheral and CNS border-associated microglia

**FIGURE 3:**
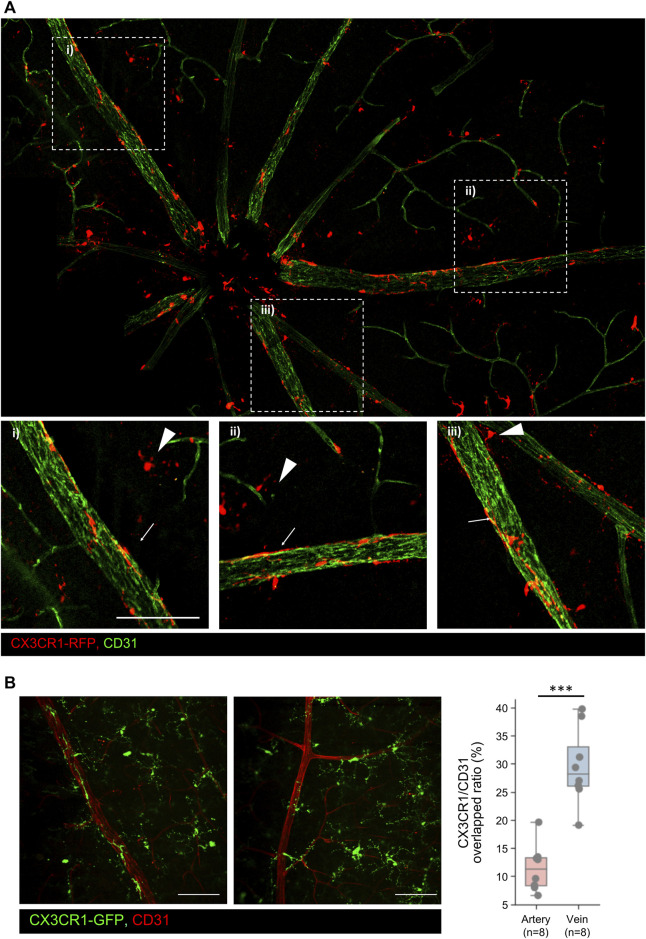
Representative images of the GCL microglia including the peripheral microglia and CNS border-associated macrophage (BAM) **(A)** Representative mosaic images of the GCL microglia including peripheral (pph) microglia and CNS border-associated macrophage. Extracted images showing typical BAMs on the retinal veins. Arrowheads indicate pph microglia, and arrows indicate the BAM. **(B)** Histological analysis of the BAM in both the veins and arteries. In addition, quantification of the CX3CR1/CD31 overlapped area ratio between the vein and artery, showing that BAM overlaps veins significantly more than arteries. n = 8/8. Scale bar: 100 μm. The data are presented as the mean ± SD. Scale bar: 100 μm; *p < 0.05, **p < 0.005, and ***p < 0.001.

In this study, we divided the GCL microglia into two subsets: pph microglia located in the retinal parenchyma and BAM lining the vascular surface of the retinal veins. Retinal BAM is unstudied compared to the brain BAM (Kim et al., 2021). Pph microglia have a typical morphology, with long processes and asterisk shapes like other microglia in the IPL and OPL; however, BAM is longitudinally lined with a stretched-form phenotype along the retinal vein from the optic nerve disc to peripheral veins, as shown in Figure 3A. BAMs are only attached to the venous surface and not to the arterial surface. Furthermore, the microglia around the vein and artery were quantified by the CX3CR1/CD31 overlapped ratio, which prominently showed a difference in the contacted surface area, even though the surface area is the combined values of the pph microglia and BAM, as shown in Figure 3B. In addition to these, there is no contribution from BAM. These perivascular-attached BAMs also increased during NaIO_3_-induced disease progression (Supplementary Figure S4). Unlike the human retina, the rodent retina has deep, penetrating veins; however, BAM is located only in the superficial retinal vein, as shown in Supplementary Video S2.

### 3.4 Specific marker for the pph microglia in the GCL microglia

To decipher the characteristics of the GCL microglia, specific markers are required for further studies. However, there are still no known markers for GCL microglia in the retina. Optimal preconditions for being specific markers include having a substance that is specifically expressed in only one cell type and being a surface protein, such as a cluster of differentiation (CD). Open accessible human data in the GEO were used for finding the microglial clusters and microglial-specific ligand–receptor pathways by using scRNA-seq and CellChat. However, most of the highly expressed RNAs released from the retinal microglia are from the cytokine family (CCL, CXCL, TNF, TGF-ɑ, IL16, and PDGF pathways) in humans; however, among these screened RNAs, galectin pathways are exclusively expressed by the retinal microglia, as shown in Supplementary Figures S8A–C (Tabel et al., 2022). Our study focuses on galectin-3 because the galectin family has many subsets, and galectin-3 is known for mediating microglial reactions in the CNS (Rahimian et al., 2018; Tan et al., 2021; Garcia-Revilla et al., 2022). Open RNA-seq data from the mice in the GEO are also used for analyzing the microglial subsets because the number of microglia in human RNA sequencing data is insufficient to be divided into subsets.

**FIGURE 4:**
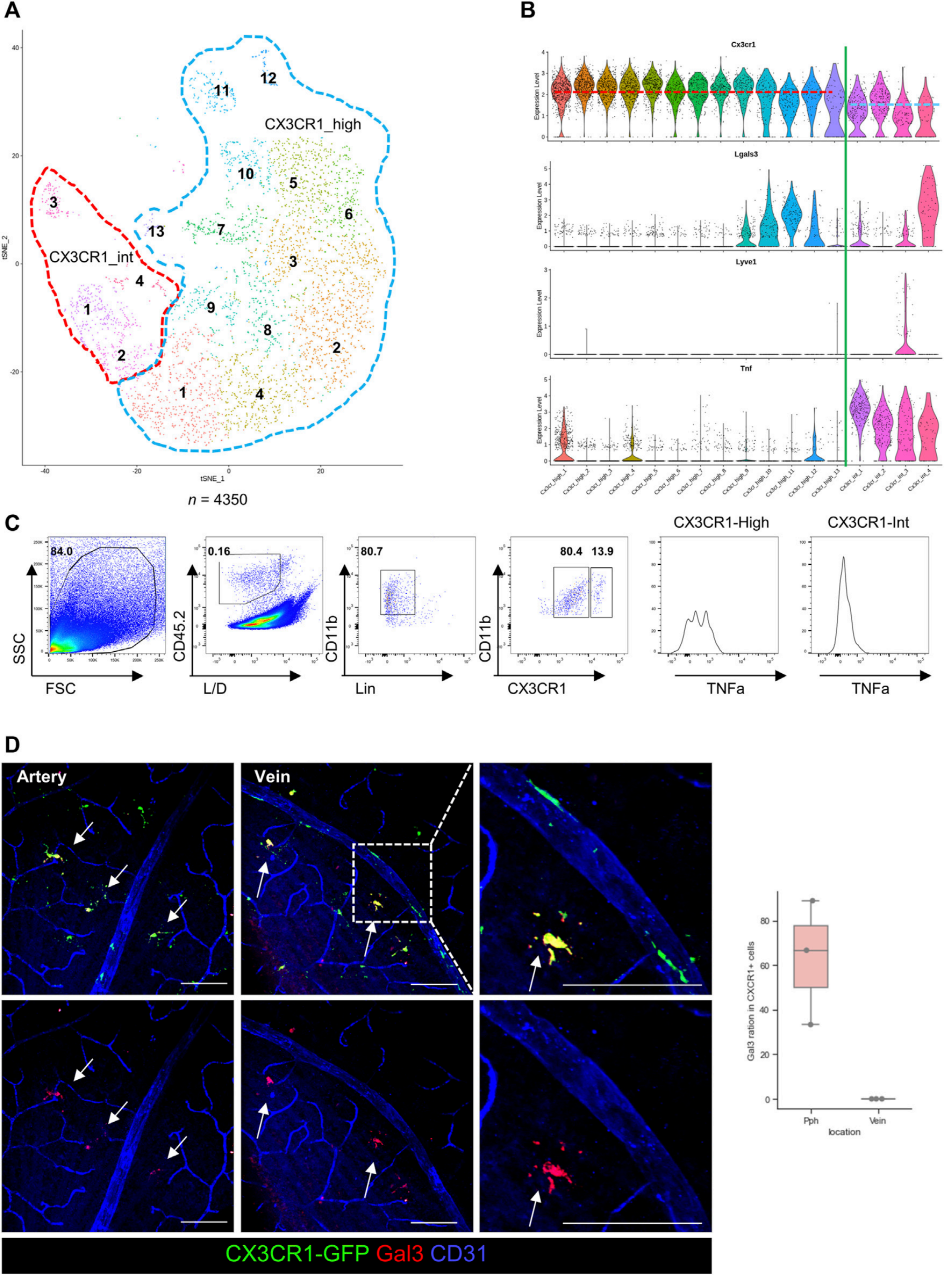
Verifications of the diversity of GCL microglia by using single-cell RNA (scRNA), FACS, and histological images. **(A)** T-distributed stochastic neighborhood embedding (t-SNE) plot of the retinal microglia in the rodent eye. These microglia are divided by the CX3CR1 expression levels, as CX3CR1-high (blue dotted line) and intermediate (int, red dotted line) groups (*n* = 4350). **(B)** The violin plots of gene expression in each microglial cluster show different gene expression patterns between CX3CR1-high and int groups. In addition, violin plots show that Lgals3 and LYVE1 genes are mainly expressed in the CX3CR1-int group. **(C)** Representative plots of flow cytometry for comparing protein expression with RNA expression from the scRNA-seq showing that RNA and protein expressions have similar patterns such as CXCR1 and TNF expression (*n* = 4 groups, each group contains 4 retinas). **(D)** Representative histological images of galectin-3 in the GCL and quantification of the galectin-3+/CX3CR1+ cell ratio between the retinal parenchyma and venous surface. The data are presented as the mean ± SD. Scale bar: 100 μm; *p < 0.05, **p < 0.005, and ***p < 0.001.

Rodent retinal microglia were pre-processed by filtering with CD45.2/CD11b/CCR2 (+/+/−) cells. A dimensional reduction plot of the pre-processed and acquired data is shown in Figure 4D (*n* = 4350). In this study, the microglial clusters were re-sorted as the CX3CR1-high expression groups and intermediate (int) groups. These groups were validated via flow cytometry by using TNF-α as a standard for CX3CR1-int groups, as shown in Figures 4D–F. Interestingly, one cluster of the CX3CR1-int group is considered a major expression cluster. Protein expression of galectin-3 in the CX3CR1-int group was also validated by a histological analysis. Galectin-3 proteins were also expressed in the CX3CR1-int cluster and only located in the retinal parenchyma, as shown in Figure 4G. Although some subsets of the CX3CR1-high cluster also express RNA of galectin-3, the surface protein expression of galectin-3 was only detected in the GCL, as shown in Supplementary Figure S5A. However, galectin-3 can act as the surface receptor in the monomer form. Otherwise, the secreted form of galectin-3 in the pentamer is a potent ligand for Toll-like receptor 4 (TLR4) signaling (Burguillos et al., 2015). Therefore, TLR4 K/O x CX3CR1-GFP-bred mice were evaluated in the same histological analysis; however, galectin-3 was only located in the peripheral retina of the GCL, as shown in Supplementary Figure S5B, which implies that galectin-3 pathways of the microglia are independent of the TLR4 signals.

**FIGURE 5:**
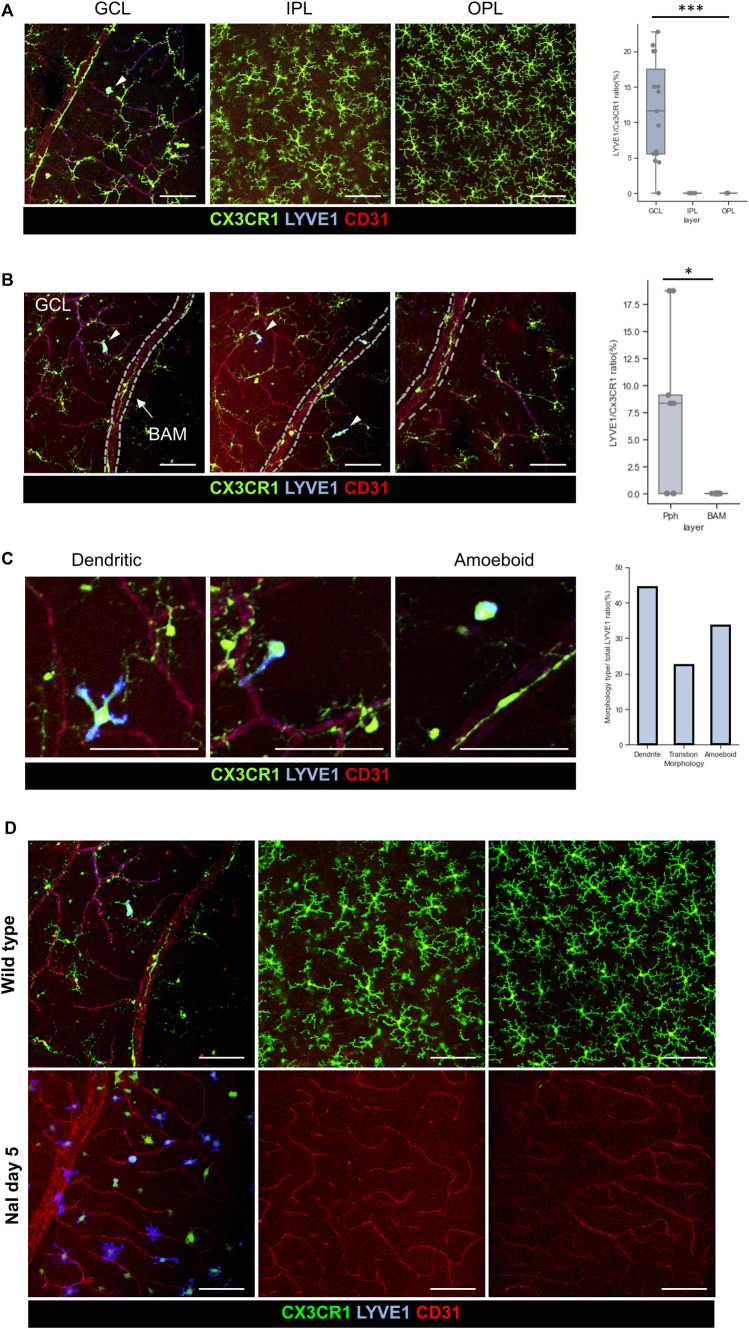
Characteristics of the LYVE1+/CX3CR1+ cells in the GCL, and accumulation of the LYVE1 expression on the GCL by using NaIO_3_ modeling. **(A)** Representative histological images of the LYVE1+ cells in each layer, and quantification of the LYVE1/CX3CR1 ratio showing that LYVE1 expression is only located in the GCL (*n* = 15/8/8 retinas). **(B)** Representative histological images including the retinal vein with BAM and quantification of the incidence of the LYVE1/CX3CR1 ratio between the pph microglia and BAM showing that LYVE1 is only expressed in the pph microglia (*n* = 9/9 retinas). **(C)** Typical morphology of the LYVE1+ microglia and quantification of each phenotype/total LYVE1 cell ratio in the healthy state (total *n* = 36 LYVE1+ cells). **(D)** Accumulation and phenotype changes in the LYVE1 cells in the NaIO_3_-induced model showing that LYVE1 is expressed and activated in the inflammatory state on the GCL surface. The data are presented as the mean ± SD. Scale bar: 100 μm; *p < 0.05, **p < 0.005, and ***p < 0.001.

Furthermore, the RNA of LYVE1 is also exclusively expressed in a subset of the CX3CR1-int group, as previously shown in the third violin plot of Figure 4E. Surface proteins of LYVE1 were also evaluated by IHC. Protein expression of LYVE1 is only located in the pph area of the GCL microglia, as shown in Figure 5A. Although LYVE1 is known as the BAM marker in the brain (Siret et al., 2022), LYVE1 was not expressed in the perivascular BAM of the proximal retinal vein (Figure 5B). LYVE1 is not expressed in fully branched resting microglia in the BAM, IPL, and OPL; however, LYVE1 is only expressed in the activated or morphologically changed microglia, as shown in Figure 5C. Under the NaIO_3_-induced disease condition, LYVE1 was prominently accumulated in the GCL, as shown in Figure 5D. Interestingly, CX3CR1 expression in some microglia with LYVE+ bulged-form processes faded. Furthermore, LYVE1 is a homolog of the CD44 and endothelial hyaluronan receptor (Banerji et al., 1999). CD44 was also evaluated, but CD44 was only expressed in the optic nerve head (ONH) of the GCL and not co-localized with CX3CR1+ cells (Supplementary Figure S6). CD44 is likely related with the astrocyte, which is abundant in the optic nerve head and known as a marker for the precursor of the astrocyte (Liu et al., 2004; Cai et al., 2012). Collectively, the CX3CR1-int group implies that the GCL microglial layer and some potential markers expressed in the GCL, such as galectin-3 and LYVE1, were coincident with the CX3CR1-int group.

### 3.5 Specific marker for BAM microglia in the GCL microglia

**FIGURE 6:**
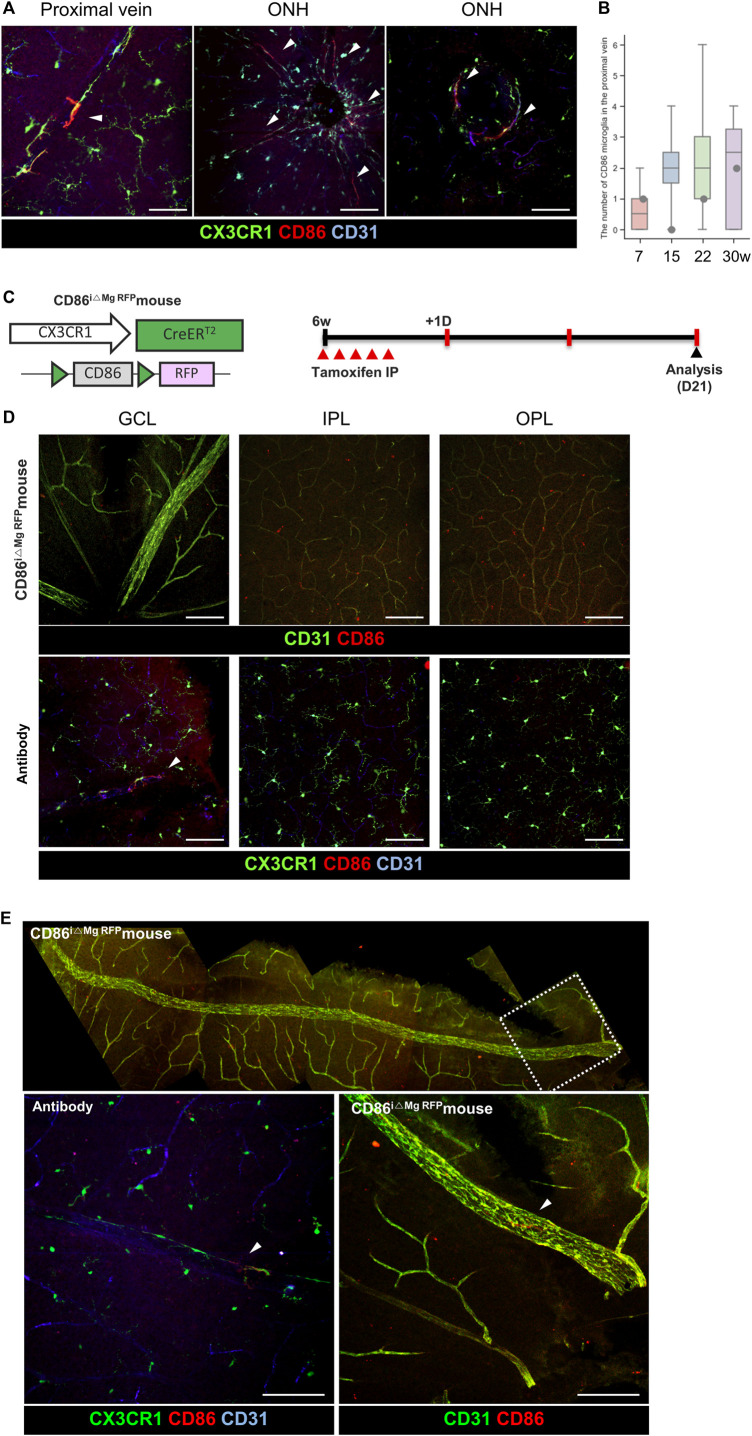
Representative histological images for minor populations of the BAM with CD86 expression around the optic nerve head. **(A)** Representative histological images of the CD86+/CX3CR1 cells around the optic nerve head and proximal retinal vein. Arrowheads indicate CD86+/CX3CR1+ cells. **(B)** Quantification of the total number of CD86 cells in the retina except the area within two disc distance (*n* = 6/4/6/8). **(C)** Schematic design of the transgenic mouse and experiment time line for visualizing the microglia-specific CD86 RFP expression. **(D)** Representative images of a CD86i△Mg RFP mouse on each layer compared with CD86 antibody-stained images showing that the soma of the transgenic mice in the all layers have CD86 expression. Arrowheads indicate CD86+/CX3CR1+ cells. **(E)** Representative mosaic images show that there is CD86 expression on the microglial processes in the proximal retinal vein. The data are presented as the mean ± SD. Scale bar: 100 μm.

A specific marker for BAM in the retina is yet to be discovered, unlike the CNS. BAMs are longitudinally distributed along the retinal vein. Potential markers for the whole BAM were screened, such as p-selectin, ICAM1, and VCAM (data not shown); however, they were not proper as whole-BAM markers. Despite this, we discovered that CD86^+^ microglia, a partial-BAM marker, is located in the BAM around the proximal vein and ONH. Except for the ONH area within two disc distances, there were a few populations of the CD86^+^ microglia on the proximal vein surfaces, as shown in Figure 6A, B, Supplementary Figure S7A. However, it seems that CD86^+^ microglia are mainly located in the ONH and partially distributed in the proximal BAMs. Transgenic reporter mice were also evaluated, as shown in Figure 6C, Supplementary Figure S7A. Dendritic expression of CD86 is only located in the proximal retinal vein; however, CD86 signals are expressed in all cell bodies of the CX3CR1 cells, as shown in Figures 6D, E, which matches with the scRNA-seq data showing that RNA expression levels are similar in each CX3CR1 group, as shown in Supplementary Figure S7B. Accordingly, it is likely that CD86 molecules are made from the nucleus and are delivered to the cell surface of the BAM processes in a certain circumstance.

## 4 Discussion

We successfully visualized the potential markers of the GCL microglia. LYVE1 and galectin-3 are used for pph microglia. CD86 is used for BAM located in the proximal retinal vein and ONH, albeit there is a report that CNS-associated microglia in the brain are mostly located around the artery (Masuda et al., 2022). Interestingly, most activated markers of the CNS, such as galectin-3 and CD86, were expressed in the GCL microglia. LYVE1 and CD44 are also expressed on the surface of the retina. Accordingly, the communication between the vitreous and retinal surfaces is important to make stable homeostasis in the posterior chamber.

The vitreous consists of two major components: hyaluronic acid and type II collagens (Tram and Swindle-Reilly, 2018). Hyaluronic acid keeps the vitreous full under young and healthy conditions; however, some diseases, aging, inflammation, and infections can result in liquefaction of the vitreous. The molecular transport between the surface of the retina and the vitreous remains unknown. LYVE1 and CD44 are hyaluronic acid receptors. LYVE1+ microglia are only located on the superficial GCL of the retina under the healthy condition, and the number of LYVE1 populations increases under disease conditions. Furthermore, the distribution of CD44 on the ONH is coincident with the main location of the astrocytes. There has been a report regarding CD44 astrocytes of the CNS (Sosunov et al., 2014), but CD44 astrocytes of the retina are still unknown. The entire LYVE1 and CD44 populations are located on the contact surface of the vitreous, even under healthy conditions, which implies that the exchange of the materials and metabolism continuously occurs. It appears that the GCL microglia are involved in vitreous remodeling through hyaluronic acid exchange. In addition, the decrease in the expression of CX3CR1 was also similar to LYVE1 perivascular macrophages of the brain (Siret et al., 2022). In addition, hyalocytes and retinal microglia share common specific markers, including CD11b, CD45, and CX3CR1 (Wieghofer et al., 2022). Retinal microglia and hyalocytes lack specific markers, making their differentiation challenging. Their distinction relies on morphological features, cell count, and responsiveness to stimulations. Despite the lower abundance of hyalocytes, their differentiation from retinal microglia remains feasible (Boneva et al., 2020; Darche et al., 2022; Wolf et al., 2022). However, the similarity in immune response patterns complicates the discrimination between these cell types. To address this challenge, the study utilized intravital or serial imaging techniques to assess morphological changes in the retinal microglia (Jeon et al., 2022). In future, experiments should be conducted to reveal the molecular mechanism of the exact roles the GCL microglia play with hyaluronic acid in the vitreous, and the specific tools for accurately distinguishing between retinal microglia and hyalocytes should be invented.

The galectin family consists of evolutionary conserved animal lectin proteins, with domains for carbohydrate recognition (Rabinovich, 1999). The conserved protein indirectly indicates that the function of galectin is necessary to maintain biological processes. Therefore, understanding the exact function of galectin in the microglia will be important to elucidate the characteristics of the GCL microglia. There have been some reports that galectin-3 of the microglia is important in the CNS, and galectin-3 has recently been the focus of many researchers in the neurodegenerative disease field (Rabinovich, 1999; Rahimian et al., 2018; Tan et al., 2021; Garcia-Revilla et al., 2022). However, knowledge about the role of galectin-3+ microglia in the retina remains limited. Galectin-3 can act as both a cytokine and surface receptor: its pentamer form acts as the cytokine, and its monomer acts as the surface receptor. The functions and pathways of galectin-3 are strongly variable; however, we found that galectin-3 has the same phenotypes on the retinal microglia by using TLR4 K/O x CX3CR1-GFP mice. Because the TLR4 pathways are major mechanisms of galectin-3, more detailed studies will be required in the future. We are still unsure if galectin-3 is located on the cell surface or cytosol. Furthermore, its roles and functions at different locations in the cell organelles must also be validated.

Conventionally, proinflammatory microglial cells are classified as M1 and are characterized by CD80, CD86, CD32, and CD11b expression (Tang and Le, 2016). Anti-inflammatory and phagocytic microglial cells are defined by M2 and characterized by CD206, arginase 1 (Arg 1), and resistin-like-α (FIZZ1) expression (Zhang et al., 2023). However, growing evidence suggests that microglial cells cannot be simply classified as M1 and M2 microglia (Ransohoff, 2016; Stratoulias et al., 2019). We previously reported on the use of CD86 as an activation marker under certain conditions such as ischemic conditions and degenerative conditions (Jeon et al., 2022; Zhang et al., 2023). However, CD86 showed slightly different phenotypes to the previously known activation markers such as CD206. In this study, we demonstrated that even under healthy conditions, the level of expression varies depending on the location of vessels. In addition, it is also well known that CD86 is a co-stimulator molecule on APCs and activation markers for microglia in the CNS. However, CD86 molecules have not been fully described in the retina. In particular, the area in which APCs come into contact with T cells has not been discovered in the retina with the intact BRB. The intact BRB blocks infiltrations of immune cells such as T cells, and the glia limitans block the penetration of inflammatory cells in the CNS parenchyma. Due to these physiological barriers, discovering the contact point between T cells and APCs in the retina has been challenging. Here, we showed that the proximal vein has a different type of APC (CD86^+^ microglia), which can potentially be the contact point between T cells and APCs in the retina. It is shown that some processes of the CD86^+^ microglia are vertically elongated in the proximal vein. We hypothesize that CD86^+^ microglia transfer immunological information inside the posterior segments to the (CTLA4+) T cells. Investigation of immunological points in the eyes will provide some evidence of this in future studies involved in neuro-inflammatory diseases. There are some limitations to our study. First, the markers suggested in this study are only valid under the healthy condition. Morphologic changes and characteristics of the microglia frequently occur when neuronal environments of the retina have been changed because reactions of the microglia depend largely on the neuronal environments. Furthermore, migration from the IPL and OPL microglia can induce disturbances in the interpretation of disease conditions. Therefore, finding unique GCL microglial markers is challenging. However, understanding the microglial characteristics under a healthy condition could help in the evaluation of future studies. Second, the mechanisms of each marker for GCL microglia should be discovered in the near future. It seems that LYVE1 and CD44 are involved in the ECM remodeling procedures on the surface of the vitreous. More detailed explanations of the exact mechanism of ECM remodeling such as hyaluronic acid coiling will be required. It is well known that immune reactions are supremely suppressed by several mechanisms in the eyes. However, it is unclear how the antigens in the vitreous are sensitized or regulated. For a subsequent study, we aim to reveal both the ECM remodeling and antigen sensitization in the vitreous. Third, there are differences between the activation markers in the brain and GCL markers in the retina. The retina and optic nerves are considered one CNS in the body. Most of the markers for microglia are initially developed for brain studies, which frequently do not work in the retina. It appears that the GCL microglia located on the surface of the vitreous are generally activated. Additional studies to find out the mechanisms of discrepancy between the brain and retina should be done in the near future.

In summary, we subdivided the retinal microglia into three layers: the OPL, IPL, and GCL. We also demonstrated that the GCL microglia have relatively low expression of the CX3CR1 gene. Subsets of the intermediate CX3CR1 groups have exclusive molecular expression such as LYVE1, galectin-3, and CD86 on the GCL. We suggested these molecules as potential markers for GCL microglia: LYVE1 and galectin-3 as pph microglia and CD86^+^ in the microglial process as BAM in the proximal retinal vein. Collectively, these molecules will help understand the CNS border reactions that have immunological and vascular heterogeneity.

## 5 Conclusion

In this work, we successfully visualized several types of retinal microglia and perivascular macrophages, as shown in Supplementary Figure S9. The GCL microglia have two subsets: pph microglia located on the retinal parenchyma and BAM, which have a special stretched phenotype only located on the surface of large retinal veins. First, in the pph microglia subset, but not in BAM, galectin-3 and LYVE1 are focally expressed. However, LYVE1 is specifically expressed in the amoeboid or transition forms, except the typical dendritic morphology in the pph microglia. Second, BAM is tightly attached to the surface of the retinal veins and has similar morphological patterns under both the healthy and disease conditions. CD86^+^ BAM has a longer process, which vertically passes the proximal retinal veins. Our data help decipher the basic anatomy and pathophysiology of the retinal microglia in the GCL.

## Data Availability

The original contributions presented in the study are included in the article/Supplementary Material, further inquiries can be directed to the corresponding authors.
