# Supplementary material for: Deciphering perivascular macrophages and microglia in the retinal ganglion cell layers
Source: Front Cell Dev Biol. 2024 Mar 26;12:1368021. doi: 10.3389/fcell.2024.1368021 (PMC11002095; doi:10.3389/fcell.2024.1368021)
Supplement: Supplementary file 3 [file DataSheet1.docx]

Supplementary Materials for

**Deciphering perivascular macrophages and microglia**

**in the retinal ganglion cell layers**

Jehwi Jeon, Yong Soo Park, Sang-Hoon Kim, Eunji Kong, Jay Kim,

Jee Myung Yang, Joo Yong Lee, You-Me Kim, In Beom Kim, Pilhan Kim

*Corresponding author. Email: pilhan.kim@kaist.ac.kr

**This PDF file includes:**

Supplementary Text

Fig. S1 to S9

**Other Supplementary Materials for this manuscript include the following:**

Supplementary Video S1 to S2

**Supplementary Fig. S1-7.**


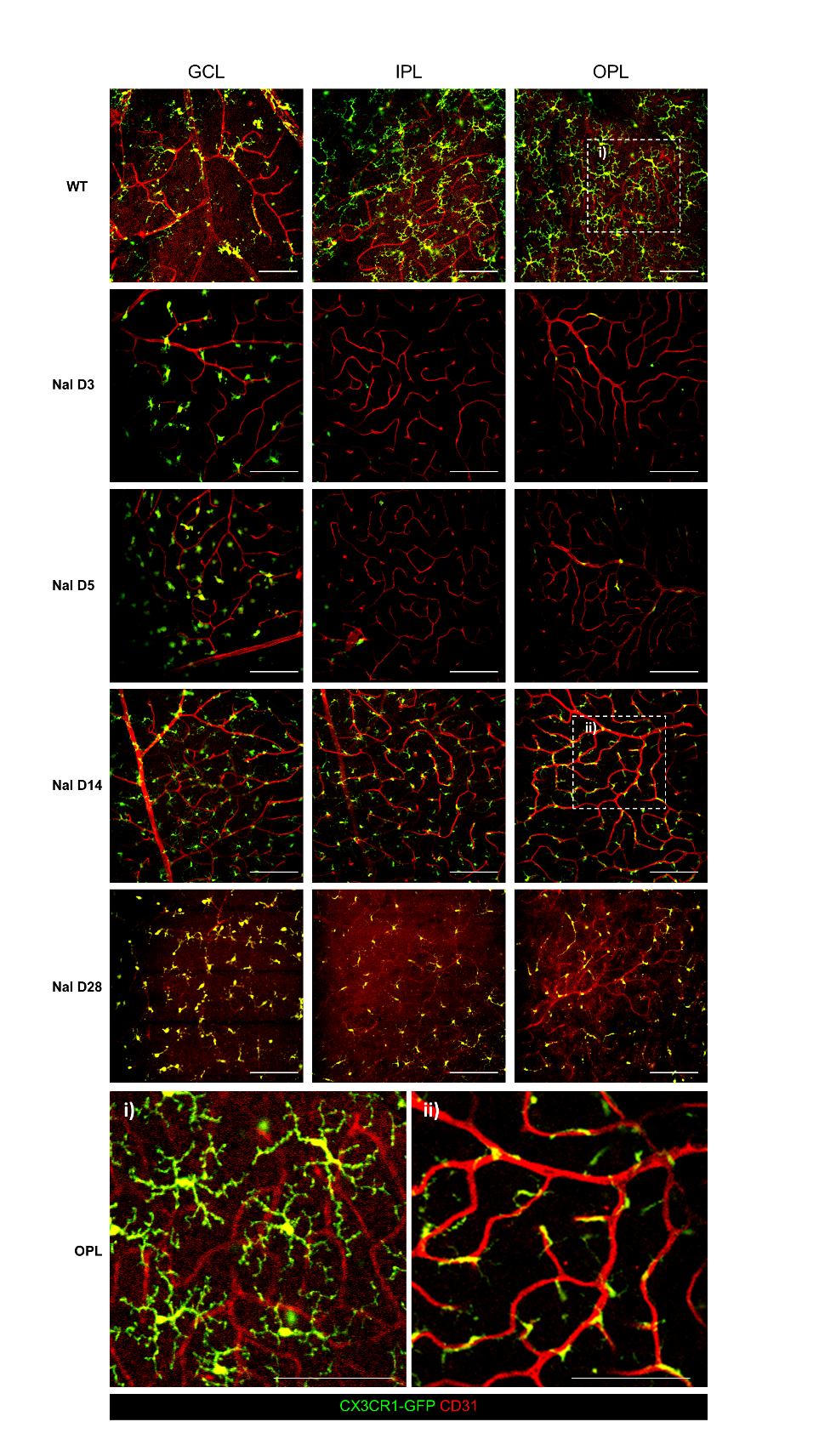


**Fig. S1. Serial immunostaining images of the NaIO3 induced RPE degeneration by using by using CX3CR1 reporter mice for 28 days.** Serial images showing that migration and accumulation of the CX3CR1+ cells during disease progression in GCL, IPL and OPL layers. These serial images show that CX3CR1+ cells are aggregated in the day 3 and 5, however, these cells are recovered and distributed in each layers after day 14. Scale bars, 100 μm**.**


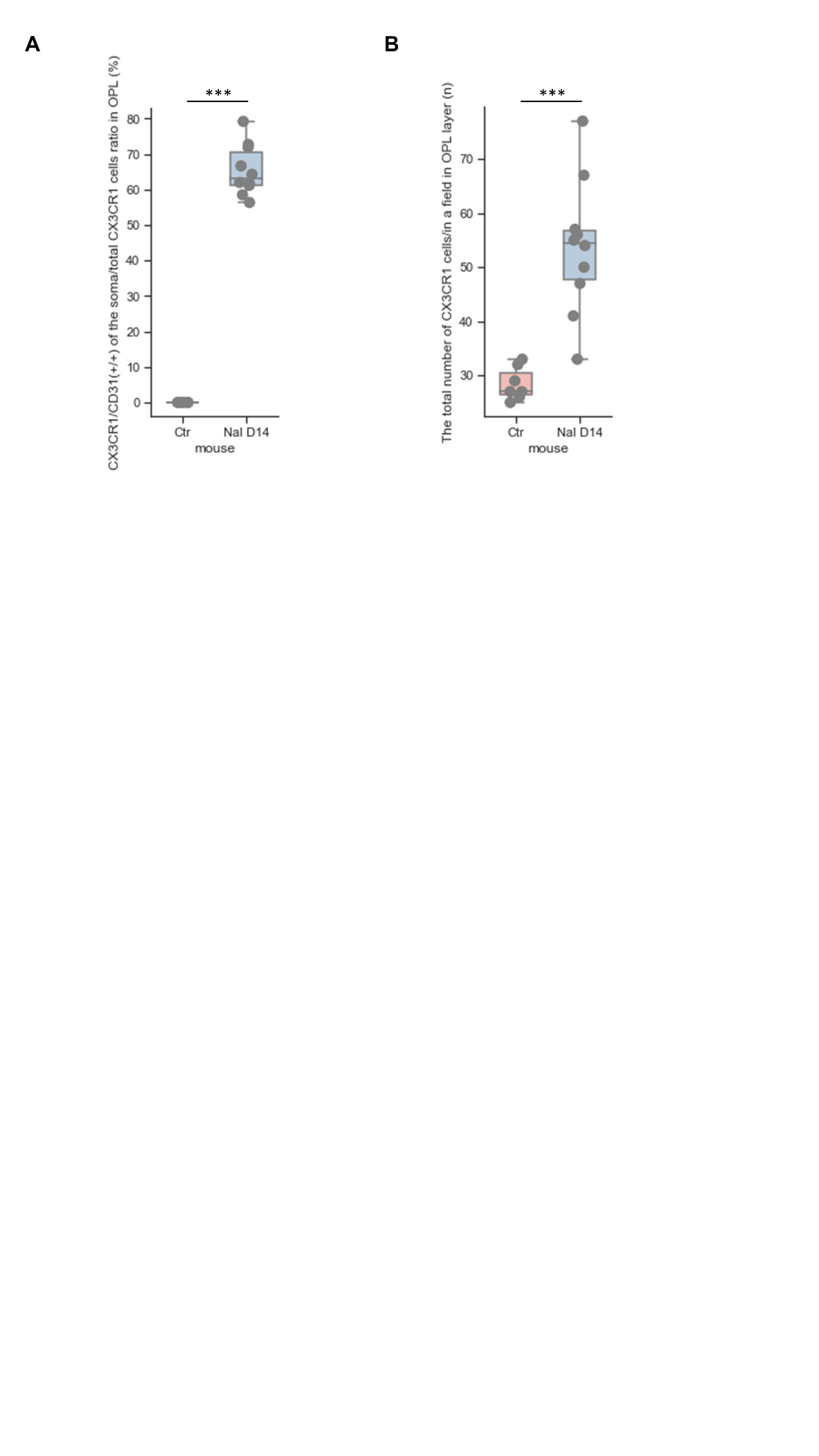


**Fig. S2. Quantifications of the CX3CR1/CD31 ratio and total cell number in the NaIO3 Day 14.** a, The ratio of the CX3CR1 cell’s soma located on the CD31+ area to total CX3CR1 cell number is calculated in the NaIO3 day 14. This graph shows that CX3CR1 cells adhere to the near capillary plexus in the disease condition. b, a box and scatter graph of the number of CX3CR1 cells infiltrated in the OPL layers. n=7/10. The data is presented as the mean ± SD. *P < 0.05, **P<0.005, ***P<0.001.


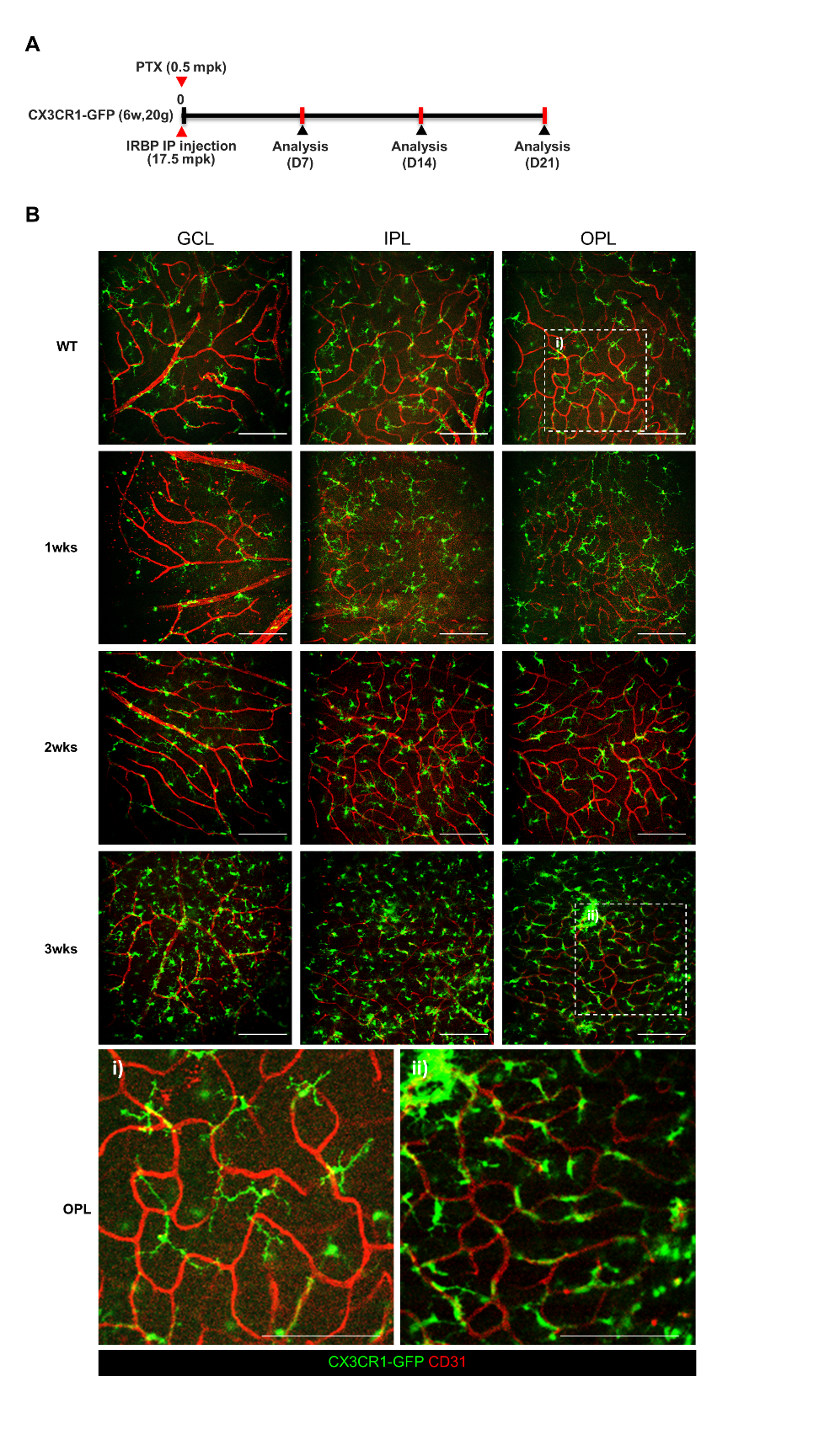


**Fig. S3. Serial immunostaining images of the IRBP induced vasculitis model by using by using CX3CR1 reporter mice for 21 days.** a, Schematic timeline of the Inter-photoreceptor retinoid binding protein (IRBP) induced autoimmune uveitis modeling in this study. b, Serial images showing that migration and accumulation of the CX3CR1+ cells during disease progression in GCL, IPL and OPL layers. These serial images show that migration and accumulation of the CX3CR1 cells to the near capillary plexus occur in the other disease models. Scale bars, 100 μm.


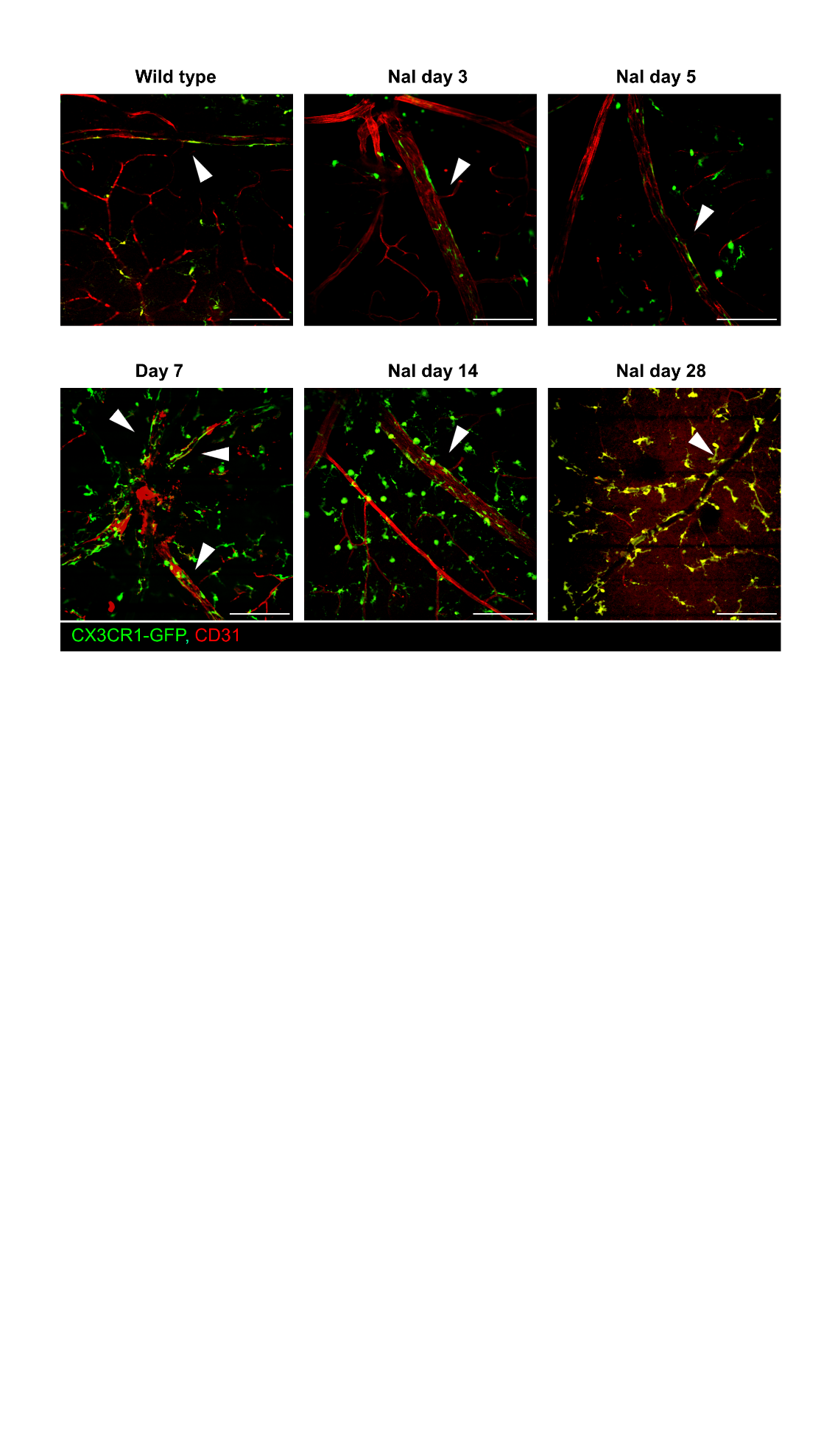


**Fig. S4. Serial immunostaining images of BAM on the proximal retinal vein in the NaIO3 induced RPE degeneration by using by using CX3CR1 reporter mice for 28 days.** Serial images show that BAM attached to surface of retinal veins are gradually increased during disease progression in the NaIO3 models. Arrowheads indicate the BAMs. Scale bars, 100 μm.


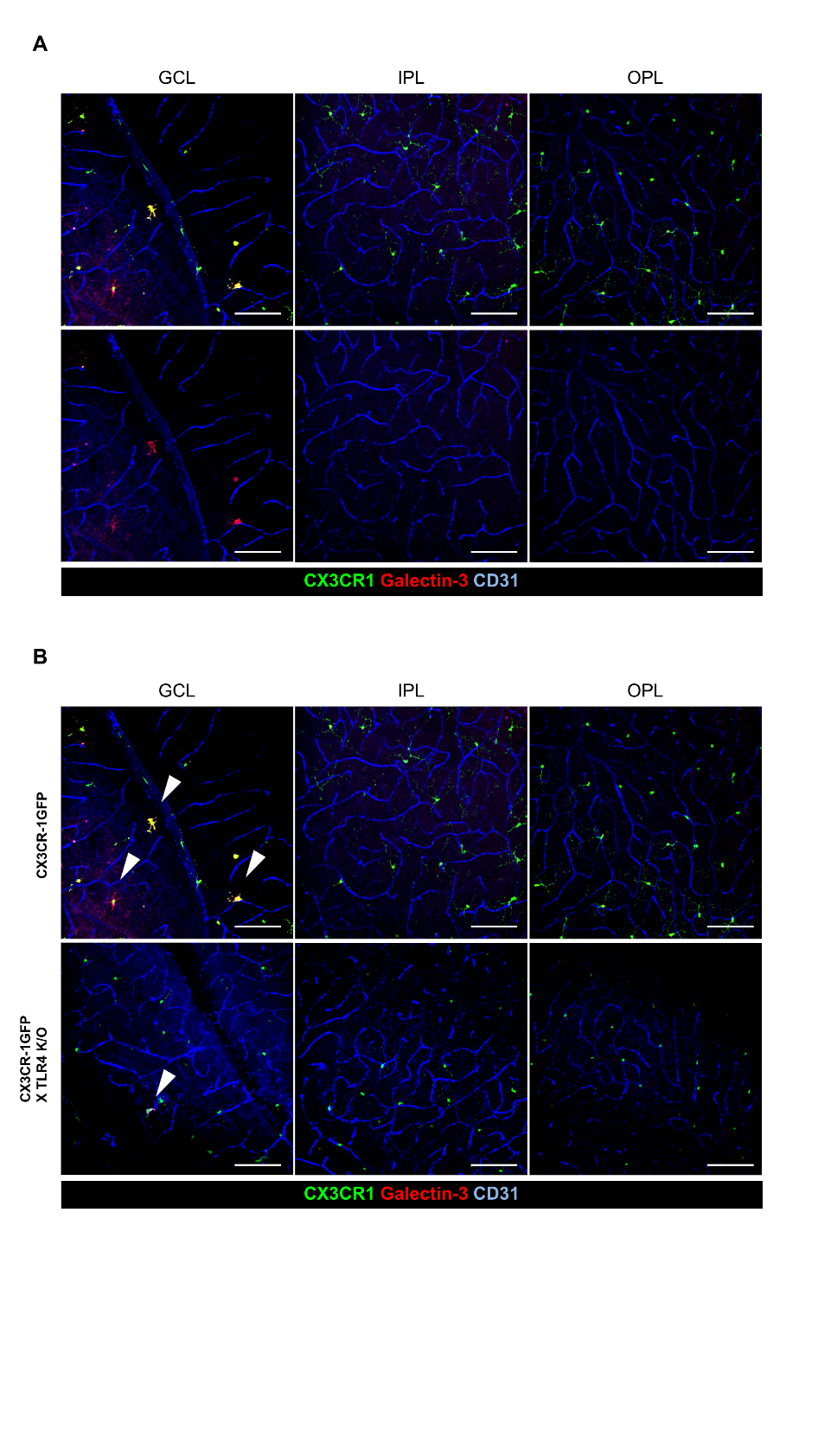


**Fig. S5. Representative histological images of Galectin-3 + microglial cells in GCL, IPL and OPL.** a, Color splitted images show that Galecitn-3+ microglial cells are only located in the GCL. b, immunostaining images of the CX3CR1-GFP x TLR4 K/O mice show that Galectin-3 + microglial cells are also detected on the GCL even in the TLR4 K/O mice. Arrowheads indicate the Galectin-3+ microglial cells. Scale bars, 100 μm.


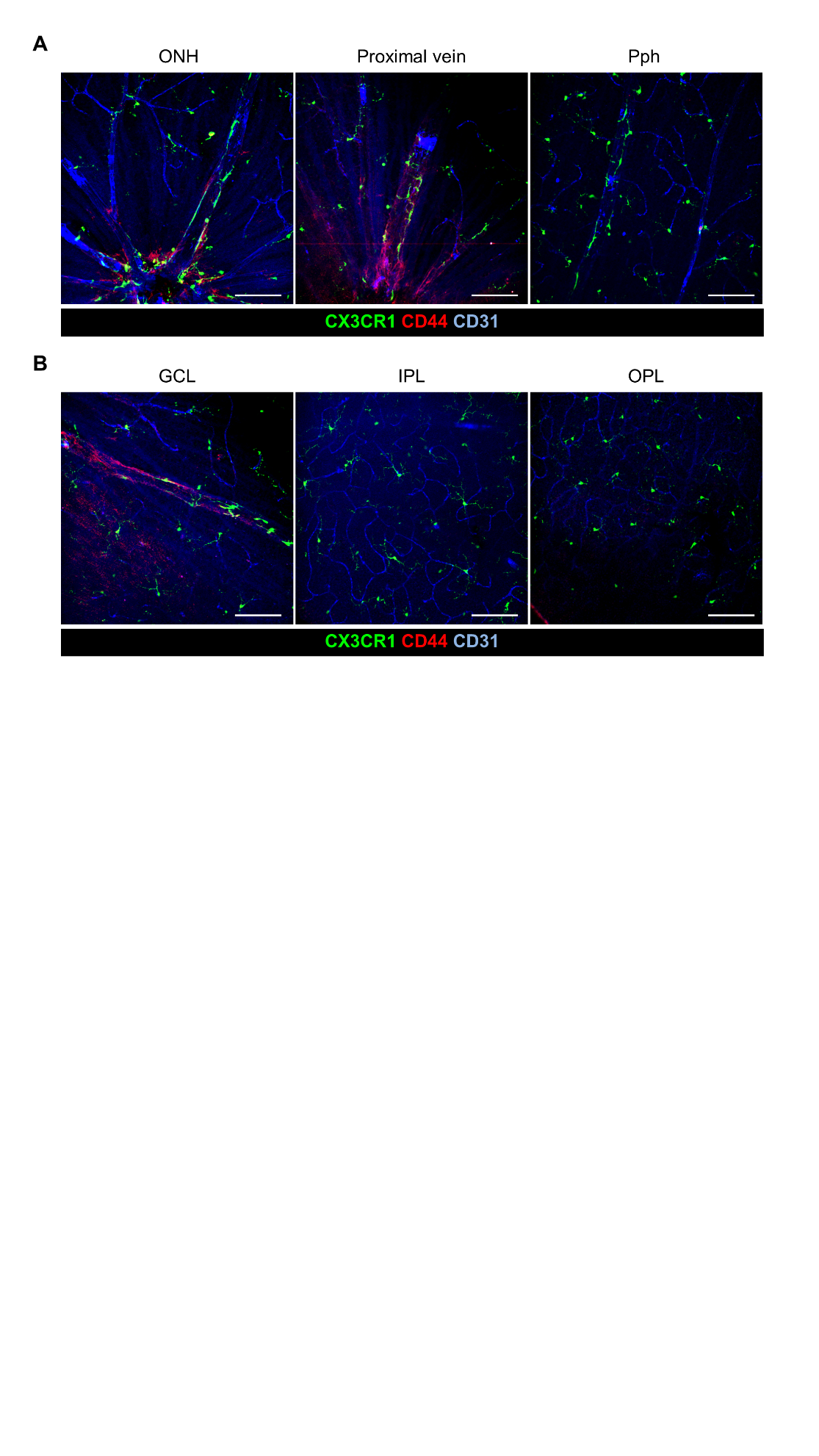


**Fig. S6. Representative histological images of CD44 around the optic nerve head.** a, histological images show that CD44+ area are located around the optic nerve head and not co-localized with the CX3CR1+ cells. b, immunostaining images show that CD44 are only expressed in the GCL layers. Scale bars, 100 μm.


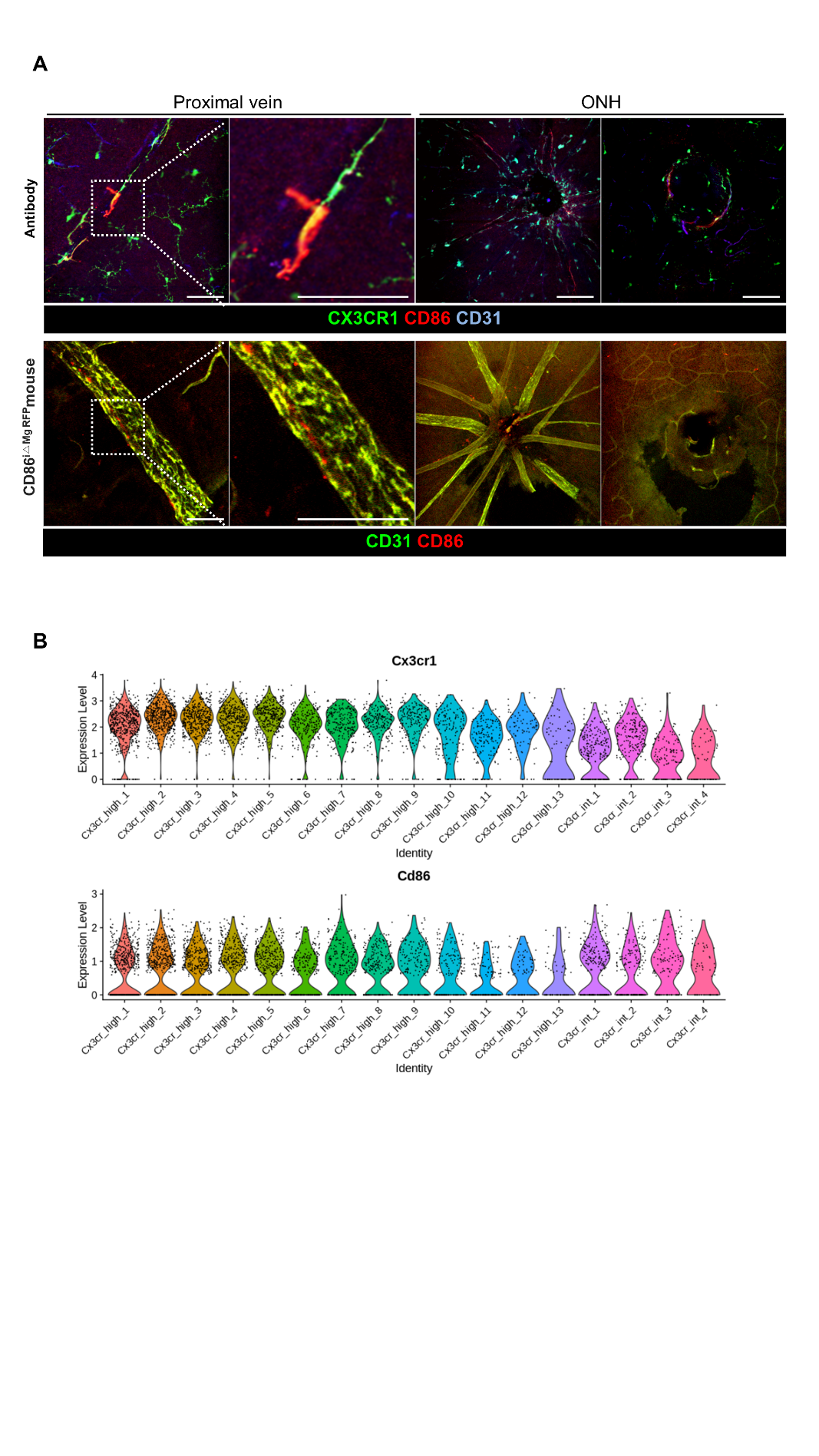


**Fig. S7. Representative histological images of CD86 around the optic nerve head.** a, histological images show that CD86+ BAM are located around the optic nerve head and proximal veins. A process of the CD86 microglia is vertically elongated to the proximal venous lumens. CD86+ microglia are mainly located in the optic nerve heads. Gray dotted boxes indicate the CD86+ BAMs. b, violin plots of the scRNA seq show that CD86 is evenly expressed in both the CX3CR1-high and int groups. Scale bars, 100 μm.


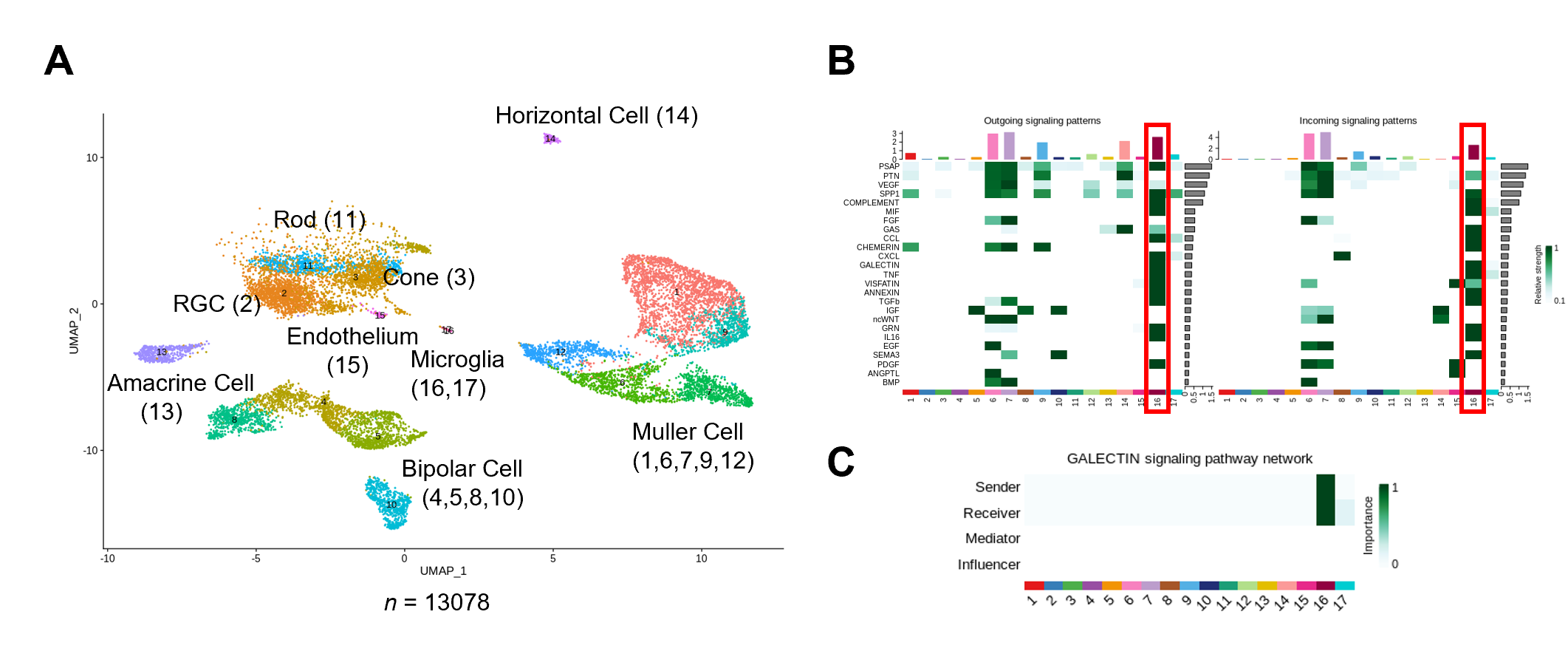


**Fig. S8. Screening for finding the GCL microglia specific markers by using scRNA seq and validation of screened potential markers via protein expressions.** a, an UMAP plot of the human major cell types in the eyes (n=13078). b, ligand-receptor signaling pathways between the human major cells. Red boxes indicate the pathways involved in the retinal microglia. c, a plot of the Galectin signaling pathway showing that the retinal microglia are exclusively involved in the Galectin pathway in the human eye.


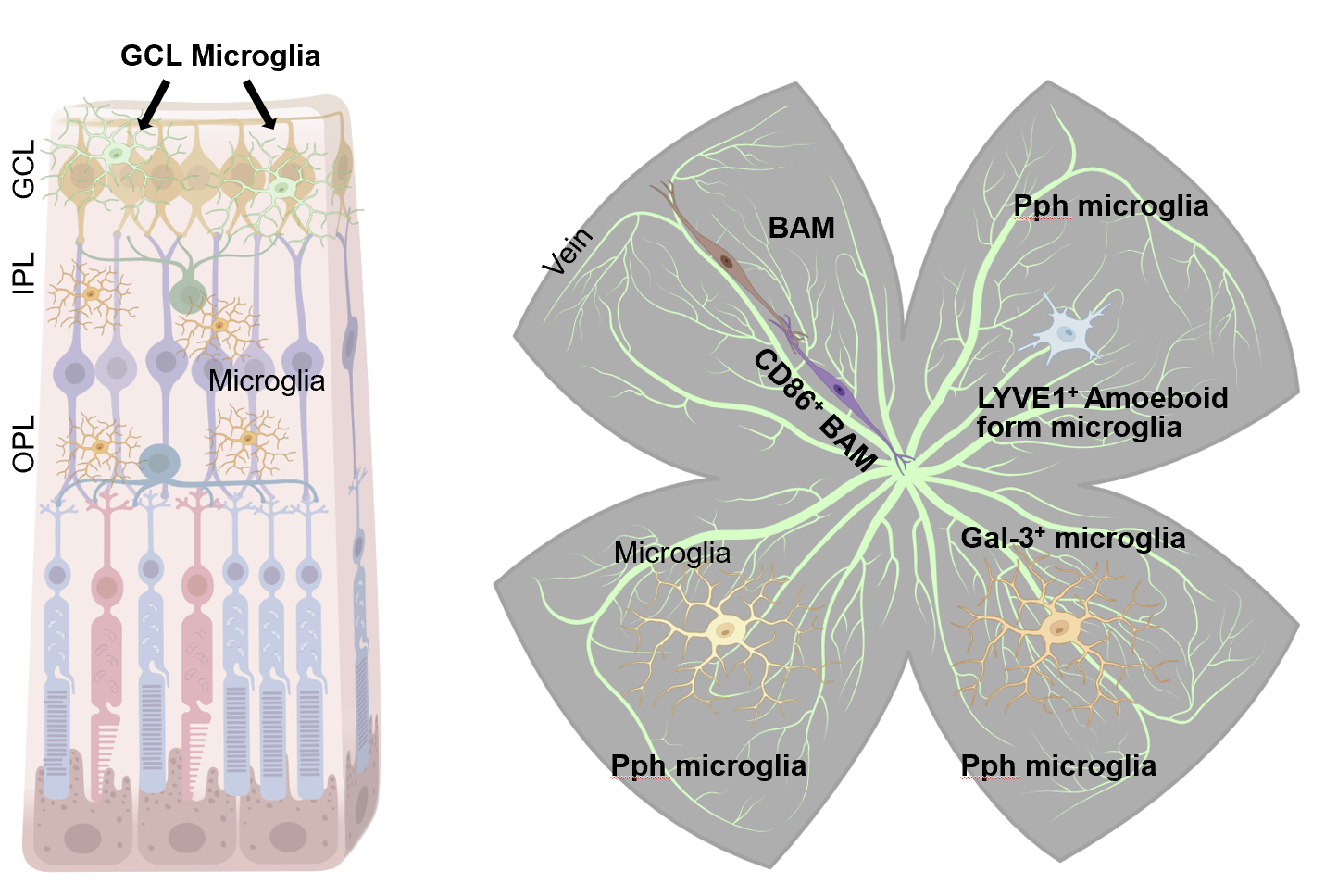


**Fig. S9. Summary illustration.** a, Vertical section of the retina showing the location of the GCL microglia and flat-mount retina indicating each covered GCL microglia in this study.

**Supplementary Video S1. 3D rendering images of inter-glial distances in the NaIO3 induced RPE degeneration model.**

**Supplementary Video S2. A 3D rendering of the superficial and deep vein with microglial attachment.**
